# Performance evaluation of 70 hepatitis B virus (HBV) surface antigen (HBsAg) assays from around the world by a geographically diverse panel with an array of HBV genotypes and HBsAg subtypes

**DOI:** 10.1111/j.1423-0410.2009.01272.x

**Published:** 2010-04

**Authors:** H Scheiblauer, M El-Nageh, S Diaz, S Nick, H Zeichhardt, H-P Grunert, A Prince

**Affiliations:** 1 Paul-Ehrlich-InstitutLangen, Germany; 2 International Consortium for Blood Safety (ICBS)New York, NY, USA, and ICBS-Europe, Düsseldorf, Germany; 3 Charité-Universitätsmedizin BerlinBerlin, Germany; 4 Hepatitis Research FoundationNew York, NY, USA

**Keywords:** HBsAg sensitivity, HBV genotypes, HBV subtypes, ICBS, S gene mutants

## Abstract

**Background and Objectives:**

This study was conducted by the International Consortium for Blood Safety (ICBS) to identify high-quality test kits for detection of hepatitis B virus (HBV) surface antigen (HBsAg) for the benefit of developing countries.

**Materials and Methods:**

The 70 HBsAg test kits from around the world were evaluated comparatively for their clinical sensitivity, analytical sensitivity, sensitivity to HBV genotypes and HBsAg subtypes, and specificity using 394 (146 clinical, 48 analytical and 200 negative) ICBS Master Panel members of diverse geographical origin comprising the major HBV genotypes A-F and the HBsAg subtypes *adw2,4*, *adr* and *ayw1-4*.

**Results:**

Seventeen HBsAg enzyme immunoassay (EIA) kits had high analytical sensitivity <0·13 IU/ml, showed 100% diagnostic sensitivity, and were even sensitive for the various HBV variants tested. An additional six test kits had high sensitivity (<0·13 IU/ml) but missed HBsAg mutants and/or showed reduced sensitivity to certain HBV genotypes. Twenty HBsAg EIA kits were in the sensitivity range of 0·13–1 IU/ml. The other eight EIAs and the 19 rapid assays had analytical sensitivities of 1 to >4 IU/ml. These assays were falsely negative for 1–4 clinical samples and 17 of these test kits showed genotype dependent sensitivity reduction. Analytical sensitivities for HBsAg of >1 IU/ml significantly reduce the length of the HBsAg positive period which renders them less reliable for detecting HBsAg in asymptomatic HBV infections. Reduced sensitivity for HBsAg with genetic diversity of HBV occurred with genotypes/subtypes D/*ayw3*, E/*ayw4*, F/*adw4* and by S gene mutants. Specificity of the HBsAg assays was ≥99·5% in 57 test kits and 96·4–99·0% in the remaining test kits.

**Conclusion:**

Diagnostic efficacy of the evaluated HBsAg test kits differed substantially. Laboratories should therefore be aware of the analytical sensitivity for HBsAg and check for the relevant HBV variants circulating in the relevant population.

## Introduction

Hepatitis B virus (HBV) is the most common, chronic viral infection globally. Approximately two billion people worldwide are affected and about 350 million have active chronic HBV infection [1,2]. In highly endemic areas such as East and Southeast Asia, sub-Saharan Africa, and parts of South America, over 8% of the population are chronic carriers of HBV [1]. 80% of adults with chronic HBV infection will have no indication that they have been infected. Due to the often silent nature of the disease, testing for HBV is imperative for public health, particularly for blood screening. Undetected acute infections and chronic carriers with low level viraemia facilitate spread of HBV. Hepatitis B surface antigen (HBsAg) is a key marker for screening and laboratory diagnosis of HBV infection and the first serological marker to appear during the course of HBV infection. HBsAg sensitivity depends on the detection threshold of immunoassays. The lower the detection limit for HBsAg, the smaller the diagnostic ‘window phase’ in early infection [3,4] and the higher the capability to detect the smallest amounts of HBsAg in asymptomatic patients and chronic carriers [5]. Thus, regulatory requirements for HBsAg are expressed as minimum analytical sensitivity to a certain HBsAg reference standard concentration [6,7]. Because of the genetic diversity of HBV, sensitivity of HBsAg assays may also be dependent on antigenic variation of HBsAg. In fact, some HBsAg mutants that emerge after selection by immune pressure can escape detection by commercial HBsAg assays [4,8–11]. In addition, there is natural heterogeneity in HBV due to genotype and subtype diversity. There are eight different HBV genotypes, A-H, based on DNA sequence. Within these are nine serological subtypes characterized by a limited number of amino acid substitutes in the ‘*a*’ determinant of the S gene, i.e., *ayw1*, *ayw2*, *ayw3*, *ayw4*, *ayr*, *adw2*, *adw4*, *adrq+* and *adrq−* [12–19]. The HBV genotypes have a distinct geographical distribution [17,18]. Genotypes A and D have global distribution, genotypes B and C predominate in East and Southeast Asia, genotype E is in West Africa, genotype F is found in the indigenous population of Central and South America, genotype G has been found in France and USA [20], and genotype H is restricted to Central and South America [15,21]. On the other hand, the standards used for calibration of the HBsAg test kits are based on genotype A subtype *ad* [22,23]. Furthermore, reduced sensitivity with HBV variants is likely to be detected only quantitatively, i.e., the immunoassay is capable of detecting them at high HBV concentrations but not at low antigen concentrations. Thus, laboratories testing blood samples for HBV are increasingly required to recognize the different HBV genotypes and subtypes and to detect very low levels of hepatitis B surface antigen. It has therefore been recommended that Regulatory Authorities devise panels for kit evaluation that include HBsAg-reactive specimens with subtypes and genotypes from their local regions [22]. To meet these needs, the International Consortium for Blood Safety (ICBS) established HBsAg Master Panels which include panel members comprising the major HBV genotypes A-F and HBsAg subtypes *adw2-4*, *ayw1-4* and *adr*. These samples were collected from blood banks around the world to evaluate HBsAg assays for sensitivity. This will assist national authorities in the developing world to make informed decisions regarding the choice of assays to be used and therefore help in establishing a sustainable supply of affordable, good quality, screening reagents.

## Materials and methods

### Specimens

Plasma units were collected by the ICBS from blood banks around the world to establish test panels that would be used to evaluate the performance of 70 HBsAg assays. The establishment of the ICBS hepatitis B virus master panels was completed in March 2005. Detailed characterization of the panels is shown in tables on the ICBS website [24]. Sequencing, genotyping, and subtyping of the panel was conducted at the Hepatitis Laboratory Branch, Division of Viral Hepatitis, Centers for Disease Control and Prevention (CDC), Atlanta, GA, as described by Purdy *et al.* [19]. The sequences, genotyping, and subtyping were also independently performed in the Department of Medical Sciences, Toshiba General Hospital, Tokyo, Japan, by the method described by Takahashi *et al.* [25]. Further characterization of the samples was done at the Paul-Ehrlich-Institut (PEI), Langen, Germany. The HBsAg content of the samples was measured by the quantitative Architect HBsAg (Abbott GmbH & Co. KG, Wiesbaden, Germany) in international units per ml (IU/ml) which also has been found to be correlated with the level of serum HBV-DNA [26]. The serological profile of the HBsAg positive samples was determined by testing for anti-HBc, anti-HBc IgM, anti-HBs, anti-HBe and HBeAg as shown on the ICBS website [24]. All samples were anti-HIV 1/2 (Architect HIV Ag/Ab, Abbott GmbH & Co. KG) and anti-HCV negative (Murex HCV v4.0, Abbott Murex Biotech Ltd., Dartford, UK and Architect HCV, Abbott GmbH & Co. KG). The large-scale fill of the ICBS Panel was done under Good Manufacturing Practice compliant conditions at the Institut für Biotechnologische Diagnostik mbH (GBD), Berlin, Germany.

### ICBS HBsAg Clinical Panel

Clinical (diagnostic) sensitivity was evaluated by the ICBS HBsAg Clinical Panel consisting of 146 HBsAg positive specimens. All samples were also anti-HBc positive by the Architect Anti-HBc assay (Abbott GmbH & Co. KG Wiesbaden, Germany). The panel is geographically diverse, including the major HBV genotypes and subtypes as displayed in Table 1 (Further information on these samples is available in Table S1 in the supporting information accessible in the online version of this article). The serological profile of the ICBS HBsAg Clinical Panel is given on the ICBS webpage [24].

**Table 1:** HBV genotypes and HBsAg subtypes in the ICBS HBsAg Clinical Panel and ICBS HBsAg Quantitative Panel

ICBS HBsAg Clinical Panel										
		Country of origin								
HBV genotype	HBsAg subtype	Brazil	Egypt	Ivory Coast	Jordan	South Africa	Tunisia	USA	Vietnam	Total no. of samples
A	adw2	12			2		2	8		24
	ayw1			5				1		6
	adw4	1								1
	ayw2					1				1
B	adw2	1						10	2	13
	ayw1							1	16	17
C	adw2	1								1
	adr							6	4	10
D	ayw2	6	1		7		6	2		22
	ayw3	1						3		4
	ayw4				1					1
	adw2		2			1				3
E	ayw4			29				1		30
F	adw4	12						1		13
Total		34	3	34	10	2	8	33	22	146
ICBS HBsAg Quantitative Panel			
Sample ID	Country of origin	HBV genotype	HBV subtype
220	Jordan	A	adw2
546	Vietnam	B	ayw1
570	Vietnam	B	adw2
516	Vietnam	C	adr
93	Tunisia	D	ayw2
318	Brazil	D	ayw3
246	Ivory Coast	E	ayw4
713	Brazil (Manaus)	F	adw4

### ICBS HBsAg Quantitative Panel

The ICBS HBsAg Quantitative Panel consists of eight HBV genotype and HBsAg subtype combinations as shown in Table 1. Amino acid alignment of the ‘a’ determinant of the S gene of the samples is shown in Table 2. The sequences were compared with the reference sequence for genotype A subtype *adw2* of Norder *et al.* [13]. A dilution series was created from each sample by preparing six HBsAg concentrations in the range of 4, 1, 0·25, 0·125, 0·063, 0·031 IU/ml using defibrinated normal human plasma as the diluent (negative for anti-HBs, HBsAg, anti-HCV anti-HIV 1/2, HCV-RNA and HIV-1 RNA). This concentration range was chosen to cover the detection limits of high and low sensitive HBsAg assays as estimated in reference [22]. The diluent served as the negative control in each dilution series. Quantification of the HBsAg concentration was done by the quantitative Architect HBsAg (Abbott GmbH & Co. KG) relative to the 2nd HBsAg WHO international standard (00/588, 33 IU/ml). Specimens with concentration values <0·05 IU/ml were considered non-reactive, and specimens with concentration values ≥0·05 IU/ml were considered reactive by the criteria of Architect HBsAg. Each dilution series of the eight genotype samples in the ICBS Quantitative Panel was linear (*r*^2^ = 1) in the given concentration range of 0·05–4 IU/ml.

**Table 2:** Amino acid alignment of the ‘a’ determinant of the S gene of the eight samples of the ICBS HBsAg Quantitative Panel

				110	120	130	140
A/*adw*		100	QGMLPVCPLI	PGSTTTSTGP	C**K**TCTT**P**AQG	NSMFPSCCCT	KPTDGNCTCI
A/*adw2*	#220	100		··········	··········	···········	···········
B/*ayw1*	#546	100	··········	···S······	·**R**········	T·········	··········
B/*adw2*	#570	100	··········	···S······	··········	T·········	··········
C/*adr*	#516	100	·········L	··TS······	·····I····	T·········	··········
D/*ayw2*	#93	100	··········	···S·······	**R**·········	T··Y······	··S·······
D/*ayw3*	#318	100	··········	···S···V···	**R**····**T**V··	T··Y······	··S·······
E/*ayw4*	#246	100	··········	···S·······	**R**····**I**···	T········**S**	··S·······
F/*adw4*	#713	100	·········L	···········	**K**····**L**···	T········**S**	··S·······
				160	170	180	190
A/*adw*		150	PIPSSWAF**AK**	YLWEWASVRF	SWLSLLVPFV	QWFVGLSPTV	WLSAIWMMWY
A/*adw2*	#220	150	··········	··········	··········	··········	··········
B/*ayw1*	#546	150	··········	··········	··········	··········	···V···I··
B/*adw2*	#570	150	··········	··········	··········	··········	···V······
C/*adr*	#516	150	·········**R**	F·········	··········	··········	···V······
D/*ayw2*	#93	150	········**G**·	F······A··	··········	··········	···V······
D/*ayw3*	#318	150	········G·	F······A··	··········	··········	···V······
E/*ayw4*	#246	150	··········	··········	··········	··········	··········
F/*adw4*	#713	150	·······LG**·**	·······A··	·······Q··	··C·······	··LV···I··

The dots represent the positions which are the same as the prototype A/*adw* sequence as referenced in [13]. Genotype and subtype specific residues according to references [14,15,18] are shown. Residues determining the genotype and subtype according to reference [19] are **bold**. D/*ayw3* substitution at positions 118 and 128 are underlined. The amino acids shown are described with one letter abbreviation according to: IUPAC-IUB Joint Commission on Biochemical Nomenclature.

### PEI HBsAg *ad* standard

The Paul-Ehrlich-Institut (PEI) HBsAg standard has been included as an independent HBsAg reference preparation which had been characterized by a biochemical, reproducible method [23]. The PEI HBsAg standard subtype *ad* has a unitage of 1000 PEI units per ml which are traceable to the 2nd WHO HBsAg international standard. One IU corresponds to 0·43 PEI units [22]. Serial dilutions of the PEI standard for HBsAg were made in fetal calf serum. The dilution range tested for each assay was from 1·0 PEI-U/ml in two-fold steps down to 0·0020 PEI-U/ml.

### ICBS Negative Panel

The Negative Panel includes 200 samples from the American Red Cross, DC, USA. All plasma units were tested and found negative for markers of HBV (HBsAg and anti-HBc), HCV (anti-HCV, HCV-RNA), HIV (anti-HIV 1/2, HIV-1 RNA), HTLV (anti-HTLV I/II), syphilis and Parvovirus B19 (DNA PCR) infection.

### Assays

All HBsAg test kits brought to the attention of ICBS were purchased from the open market and not directly from the manufacturers. This was to ensure that test kits chosen for evaluation were from routine production runs, thus avoiding any possible biased selection by manufacturers for batches of higher sensitivity that might not reflect the usual performance of the assay.

The 70 HBsAg assays evaluated included 51 enzyme immunoassays (EIA) and 19 rapid assays. Information regarding the assays used in this study including product name, manufacturer, test format, test structure, catalogue numbers, and manufacturers’ contact information are shown on the ICBS website [24].

### Laboratory testing

Starting June 2005 the 70 HBsAg test kits were evaluated, in the order they were received, by the ICBS Test Kits Evaluation Centre at the Paul-Ehrlich-Institut (PEI) in Langen, Germany. The assays were carried out and interpreted according to the instructions for use provided by each manufacturer. One person carried out all the testing for a single assay. Specimens discordant from the assigned pedigreed status were repeated in duplicate and the repeat reactive rate was taken for sensitivity and specificity calculation. With the rapid assays, discordant results were read independently by a second person. Where the two readers disagreed, the test result was considered equivocal.

### Calculation of analytical sensitivity, diagnostic sensitivity and specificity

For determination of the analytical sensitivity, the intercept of the dilution series with the assay’s cut-off point was calculated by linear interpolation between the last positive and the first negative point. Also, conversion of the assay’s results into IU/ml was done by linear interpolation. The results for the PEI HBsAg *ad* standard and for the ICBS Quantitative Panel were expressed in IU/ml relative to the 2nd WHO HBsAg international standard. Sensitivity reduction for the various genotypes and subtypes was calculated for each assay as a factor relative to the result for genotype A subtype *adw2* in the same assay. Diagnostic sensitivity and specificity were calculated according to standard procedures [27,28]. To summarize, diagnostic sensitivity was calculated as the number of HBsAg positives of an assay divided by the total number of HBsAg confirmed positive panel specimens tested, multiplied by 100. Test specificity was calculated as the number of HBsAg negatives found by an assay and divided by the total number of confirmed HBsAg negative panel specimens tested, multiplied by 100.

### Correlation of HBsAg kinetics in seroconversion with analytical sensitivity for HBsAg

Thirty-two commercially available HBV seroconversion panels were analysed for the kinetics of HBsAg concentration and for the time needed to detect specified HBsAg concentrations of 0·02, 0·05, 0·13, 0·5, 1·0 and 4·0 IU/ml. The panels were from SeraCare Life Science (West Bridgewater, MA) and Zeptometrix Inc. (Franklin, MA). The HBsAg concentrations of the panel members were determined with the Architect HBsAg (Abbott GmbH & Co. KG) and the PRISM HBsAg (Abbott). To describe the kinetics of HBsAg detection, an exponential function (exp(a*t−b)), where t = day, was fitted to each of the 32 seroconversion panels (procedure ‘nls’ from R) [29]. To estimate the time until detection of the pre-specified HBsAg levels, the inverse of the fitted regression function was used.

## Results

### Analytical sensitivity of the 70 HBsAg test kits

**Fig. 1:**
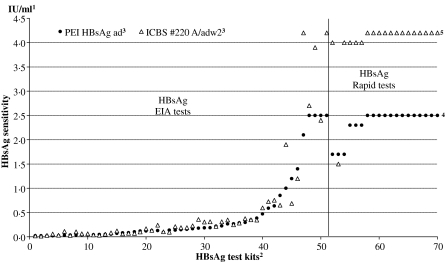
Analytical sensitivities of the 70 HBsAg test kits evaluated. ^a^Analytical Sensitivities for each assay are expressed as IU/ml relative to the 2nd HBsAg WHO international standard. ^b^The 70 HBsAg test kits were sorted according to their analytical sensitivity with the PEI HBsAg ad standard. ^c^Each symbol in figure represents the detection limit of an HBsAg kits as shown in Table S2 in the electronic version of the article. ^d^Detection limits > 2·3 IU/ml are shown as 2·5 IU/ml.

**Fig. 2:**
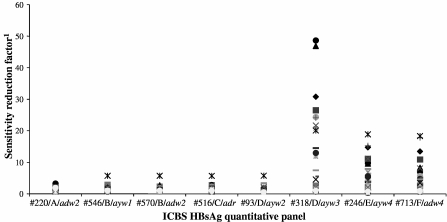
Sensitivity differences of the HBsAg EIAs for the various HBsAg genotypes A–F and subtypes adw2/4, adr and ayw1–4 in the ICBS HBsAg Quantitative Panel. ^a^Sensitivity reduction factors are expressed for each assay relative to the analytical sensitivity for genotype A subtype *adw2* sample #220 in the same assay. The symbols in figure represent the sensitivity reduction factor of the different HBsAg EIA kits (*n*= 51). The values of figure refer to Table S2 in the electronic version of the article. The rapid tests were not included because they do not give continuous values.

Figure 1 shows the range in analytical sensitivities of the 70 HBsAg tests with the ICBS HBsAg Quantitative Panel genotype A subtype *adw2* and the PEI HBsAg standard subtype *ad* (1000 PEI-U/ml). The range of sensitivity between the most sensitive test kit and the least sensitive HBsAg devices was 0·021 IU/ml to >2·33 IU/ml with the PEI *ad* standard and 0·013 IU/ml to >4 IU/ml with the ICBS Quantitative Panel. This represents a >300-fold difference between the most sensitive assay and the least sensitive devices. Twenty-three HBsAg test kits had analytical sensitivities <0·13 IU/ml, which represents the current state of the art for blood screening in the European Union (EU) [3,4,6]. Twenty EIAs were between 0·013 IU/ml and 1 IU/ml, seven EIAs had sensitivities >1 IU/ml, and one EIA could not detect any of the HBsAg concentrations tested (>4 IU/ml). Regarding the 19 HBsAg rapid assays tested, one could detect 1·5 IU/ml, two rapid assays had sensitivities of 1·7–4 IU/ml, three rapid assays barely detected 4 IU/ml, and 13 rapid assays did not detect any of the ICBS standard dilutions as positive, i.e., these tests can only detect undiluted specimens with HBsAg levels of over 4 IU/ml (The detailed results are shown in Table S2 in the supporting information accessible in the online version of this article). Figure 1 also shows that in the assays evaluated the ICBS genotype A subtype adw2 (panel #220) correlated well with the PEI HBsAg ad standard. The sensitivity of the eight different HBV geno-/subtypes in the different assays relative to genotype A subtype *adw2* for the 51 EIAs is displayed in Fig. 2. Thirty test kits showed even sensitivity for all genotypes/subtypes studied, 17 HBsAg test kits showed reduced sensitivity concomitantly for the genotypes/subtypes D/*ayw3*, E/*ayw4* and F/*adw4*, one assay showed sensitivity reduction for genotype/subtype D/ayw3 and another assay showed reduced sensitivity for the genotypes/subtypes E/*ayw4* and F/*adw4* only. The rapid assays did not give continuous values and were not included in Fig. 2 but one rapid assay also showed the pattern of concomitant sensitivity reduction for genotypes/subtypes D/*ayw3*, E/*ayw4* and F/*adw4.* Seventeen rapid assays and four EIAs were too insensitive in the range of the dilution series tested (≤4 IU/ml) to judge for quantitative differences. The degree of the geno-/subtype dependent sensitivity reductions ranged from 4 to 50 for D/*ayw3* and from 2 to 20 for both, E/*ayw4* and F/*adw4*. In case of the sensitivity reductions occurring, singly for D/*ayw3*, or for E/*ayw4* and F/*adw4*, the sensitivity reduction ranged from 2 to 3.

### Correlation of HBsAg kinetics in seroconversion with analytical sensitivity for HBsAg

**Fig. 3:**
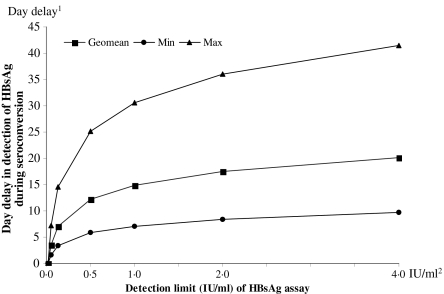
Correlation of analytical sensitivity of HBsAg assays with the time needed for detection of HBsAg. ^a^Day delay relative to the most sensitive HBsAg assay (0·02 IU/mL). ^b^IU/mL relative to the 2nd WHO International HBsAg Standard (00/588). Geomean, geometric mean value of 32 seroconversion panels tested; min/max, the smallest and largest number in the set of values. For the values of figure refer to Table S3 in the electronic version of the article.

The HBsAg concentration increases exponentially with the time of the follow-up samples during seroconversion. Inversely, the time (days) needed for detection of HBsAg during seroconversion increases logarithmically with the detection limit for HBsAg. This is shown in Fig. 3 averaged over the 32 seroconversion panels tested (The detailed results are available in Table S3 in the supporting information accessible in the online version of this article). The day delay in HBsAg detection at specified assays′ analytical sensitivities was compared to the first positive HBsAg detection at 0·02 IU/ml. By this, HBsAg detection limits of 0·05 IU/ml, 0·13 IU/ml, 0·5 IU/ml, 1 IU/ml, 2 IU/ml, and 4 IU/ml correspond to a mean day delay in detection of HBsAg assays of 3·5 (range 1·6–7·2), 7·1 (range 3·4–14·6), 12·2 (range 5·9–25·1), 14·8 (range 7·1–30·6), 17·5 (range 8·4–36 IU/ml), and 20·1 (range 9·7–41·4) days, respectively. Relative to an HBsAg detection at 0·05 IU/ml, corresponding to the Architect HBsAg (Abbott), this represents a mean day delay of 3·6 (range 1·8–7·4), 8·7 (range 4·3–17·9), 11·3 (range 5·5–23·4), 14 (range 4·9–32·5) and 16·6 (range 8·1–34·2) days, respectively.

### Diagnostic sensitivity with the ICBS HBsAg Clinical Panel

Diagnostic sensitivity with the ICBS HBsAg Clinical Panel in the 70 test kits evaluated is shown in Table 3 (Further information is available in Table S4 in the supporting information accessible in the online version of this article). Eighteen EIA test kits were 100% positive, the other 33 EIAs were false negative in 1–4 samples (sensitivity range 99·3–97·3%), and the 19 rapid assays had 2–8 false negatives (sensitivity range 99·3–94·5%). The false negatives were mainly with the ICBS Clinical Panel samples #1125, #1135, #1010, #1039 and #1015. This was seen with some HBsAg test kits even if the detection limit actually should have been able to detect the HBsAg concentration in these samples: Sixteen test kits were negative with at least one of the samples #1125, #1135, and #1010, two EIAs and two rapid assays failed to detect HBsAg in sample #1039 E/*ayw4* (72 IU/ml), and four rapid assays did not detect sample #1015. Other HBsAg test kits were positive with the same five samples but showed significantly reduced sensitivity. Table 3 shows the reduction factors. There was 20-fold reduced sensitivity with mutant T131I (#1010), 13- to 120-fold reduced sensitivity with mutant Q101H (#1039), and mutant S143L (#1015) revealed a marked 500- to >1000-fold sensitivity reduction in two EIAs which would have been negative if it had not been for the high HBsAg concentration in the sample (>8000 IU/ml). Moreover, some rapid assays revealed deficiencies in detection of more samples. One rapid assay was false negative with samples #1105 B/*ayw1* (19·16 IU/ml), #1121, B/*adw2* (35·14 IU/ml), #1115 B/*adw2* (50·77 IU/ml), and #1106 B/*ayw1* (50·97 IU/ml). Another rapid assay did not detect sample #1032 E/*ayw4* (21·91 IU/ml). Four other rapid assays were equivocal with sample #1105 B/*ayw1* (19·16 IU/ml).

**Table 3:** Relative sensitivity reduction in HBsAg assays with the problematic samples of the ICBS HBsAg Clinical Panel

			ICBS HBsAg Clinical Panel samples						
			Sample ID		#1125	#1135	#1010	#1039	#1015
			Genotype/subtype		B/adw2	B/adw2	D/ayw2	E/ayw4	D/ayw2
			Mutation		M133L	P105R	T131I	Q101H	S143L
			HBsAg concentration (IU/ml)		0·21	0·22	0·36	72	>8000

Test format	Number of assays (*n*)	Clinical sensitivity (%)	Analytical sensitivity (IU/ml)^a^	Number of false neg. (*n*)	Relative sensitivity reduction^b^ (*n*/*n*)^c^				
EIA	18	100	0·02–0·13	0	0	0	0	0	500 to >1000 2/18
EIA	5	99·32–98·63	0·07–0·13	1–2	1–25 4/5	1–25 4/5	0	0	20 1/5
EIA	9	99·32–98·63	0·14–0·22	1–2	1–25 6/9	1–25 7/9	0	0	0
EIA	6	98·63–97·26	0·23–0·36	2–3	BDL 4/6	BDL 6/6	20 1/6	120	0
EIA	13	98·63–97·26	0·39–2·3	2–4	BDL 12/13	BDL 12/13	BDL 11/13	13–120 2/13	0
Rapid	19	98·63–94·52	1·7–2·3	2–8	BDL 19/19	BDL 19/19	BDL 18/19	2/19	4/19

Detailed data is available in Table S4 in the electronic version of this article.

a Detection limit see Table S2.

b Approximated relative factor in sensitivity reduction compared to ICBS A/adw2 (panel #220).

c Number of assays reacting with sensitivity reduction out of total assays in the line.

BDL, below detection limit.

### Amino acid alignment of the ICBS HBsAg Quantitative Panel members and of the HBsAg mutant samples in the ICBS HBsAg Clinical Panel

The amino acid alignment of the S gene of the eight genotype/subtype samples of the ICBS HBsAg Quantitative Panel is shown in Table 2. The primary structure for the various genotypes is within the expected range of the reference sequences [13,16], but genotype/subtype D/*ayw3* sample #318 shows a particular A118V/T128V double substitution characteristic for a specific D/*ayw3* strain [14]. Genotypes/subtypes D/*ayw3*, E/*ayw4*, and F/*adw4*, which were detected with reduced sensitivity in some assays, share a substitution at position 127, i.e., P127T for the *ayw3* subtype and T127I/L specific for E/*ayw4* and F/*adw4*. Moreover, E/*ayw4* and F/*adw4* have the genotype specific P140T residue.

Amino acid alignment of the S gene of the five samples of the ICBS Clinical Panel which caused reduced sensitivity in some test kits revealed the following mutations in the ‘*a*’ determinant as shown in Table 4: M133L in sample #1125, P105R in sample #1135, T131I in sample #1010, T/S143L in sample #1015, and Q101H in sample #1039.

**Table 4:** Amino acid alignment of the HBsAg mutant samples of the ICBS HBsAg Clinical Panel which caused reduced sensitivity in some HBsAg test kits

			110	120	130	140	150
A/*adw2*			QGMLPVCPLI	PGSTTTSTGP	CKTCTTPAQG	NSMFPSCCCT	KPTDGNCTCI
B/*adw2*	#1125	100	··········	··········	··········	T·**L**·······	··········
B/*adw2*	#1135	100	····**R**···I·	··········	··········	T·········	··········
D/*ayw2*	#1010	100	··········	···S······	·R········	**I**··Y······	··S·······
D/*ayw2*	#1015	100	··········	···S······	·R········	T··Y······	··**L**·······
E/*ayw4*	#1039	100	·**H**········	····S·····	·R····L···	T········S	··S·······
			160		170
A/*adw2*			PIPSSWAFAK	YLWEWASVRF	SWLS
B/*adw2*	#1125	150	·········K	F·········	····
B/*adw2*	#1135	150	·········K	··········	····
D/*ayw2*	#1010	150	········GK	F······A··	····
D/*ayw2*	#1015	150	·········K	F······A··	····
E/*ayw4*	#1039	150	········GK	F······A··	····

The dots represent the positions which are the same as the sequence in reference [13] but the variations specific for the respective genotypes according to reference [13] are shown. Mutations are shown in **bold** and underlined. The amino acids shown are described with one letter abbreviation according to: IUPAC-IUB Joint Commission on Biochemical Nomenclature.

### Specificity

Specificity in the ICBS Negative Panel of 200 negative samples was 100% in 50 assays, seven assays showed one non-specific result with specificity of 99·5%, and nine assays showed more than one non-specific: 99·0% (*n* = 4), 98·5% (*n* = 1), 97·0% (*n* = 2) and 96·4% (*n* = 2). Four assays, though ordered, were not supplied in sufficient quantity to determine specificity.

## Discussion

The results of the performance evaluation of 70 HBsAg test kits show that the diagnostic efficacy of the tests differed significantly. The sensitivity range between the most sensitive HBsAg devices and the least sensitive HBsAg assays was more than 300-fold. Enzyme immunoassays (EIA), in general, performed better than rapid assays. However, also within the EIAs there was a significant 200-fold variation in sensitivity; moreover, five EIAs were less sensitive than rapid assays. Combining the results for analytical sensitivity, clinical sensitivity, and sensitivity for HBV variants, resulted in the following overall performance picture. A group of 17 assays showed high analytical sensitivity of <0·13 IU/ml comparable to the level of current blood screening tests in the EU [3,4,8], were 100% sensitive in the ICBS Clinical Panel, and were even sensitive for all HBV variants tested. A second group of 6 EIA test kits showed also high sensitivity of <0·13 IU/ml [8]. However five of these six assays missed HBsAg mutants in the ICBS Clinical Panel, two of which also showed significant reduced sensitivity for genotypes D/*ayw3*, E/*ayw4* and F/*adw4* in the ICBS Quantitative Panel, and another assay showed genotype dependent sensitivity reduction only. A third group of 20 HBsAg EIAs kits was in the sensitivity range of 0·13–1 IU/ml, but did not detect all clinical samples at HBsAg concentrations of 0·21–0·36 IU/ml due to mutations in the S gene and/or because of low sensitivity. Furthermore, 16 assays in this group also showed significant reduced sensitivity for HBV genotypes/subtypes D/*ayw3*, E/*ayw4* and F/*adw4*. A fourth group of eight EIA HBsAg tests and all 19 rapid assays studied were of poor sensitivity (>1 IU/ml). Thirteen rapid assays and one EIA of the third group did not detect any of the dilution series at 4 IU/ml; and 5 of these 13 rapid assays revealed difficulties in detecting HBsAg concentrations around 20 IU/ml in the ICBS Clinical Panel.

Correlation of HBsAg seroconversion kinetics with analytical assay sensitivities reveals that the detectable HBsAg positive period at detection limits of 1 IU/ml and 4 IU/ml is reduced to levels not deemed suitable for screening blood donations. The delay in detection of HBsAg at analytical sensitivities of 1 IU/ml and 4 IU/ml averages to 11·3 days and 16·6 days, respectively, relative to an HBsAg assay detection limit of 0·05 IU/ml, which reflects the current blood screening standard, e.g. in the EU. Assuming a similar delay in HBsAg detection in the peak-off viremia of HBV, this would correspond to a total reduction of the detectable HBsAg positive period of 22·6–33·2 days, respectively following HBV infection. Based on a total detectable HBsAg EIA positive period of 31 days in asymptomatic patients (ALT < 100 IU/ml) and of 82 days in symptomatic (ALT > 100 IU/ml) HBV infection [30] this means virtually no detectable HBsAg in asymptomatic HBV infection and a considerable shortening of the detectable HBsAg period of 82 days in symptomatic HBV infection [30]. This would make it also improbable that low level HBsAg is reliably detected positive in late resolving phase and chronic carriers. With respect to the rapid tests in general, it appears that further development is necessary to achieve acceptable sensitivity for blood screening [31].

Influence of HBV genotype and HBsAg subtype variability on HBsAg sensitivity was seen simultaneously, for D/*ayw3*, E/*ayw4*, and F/*adw4,* in a total of 18 test kits. A common motif in these three genotypes/subtypes seems to be represented by the genotype specific residue 127 where Proline (P) is changed by Threonine (T) in D/*ayw3* and by Leucin (L) in E/*ayw4* and F/*adw4*. In addition, the D/*ayw3* sample #318 includes the 118/128 double mutation, characteristic for a naturally occurring strain distributed worldwide [14,31,32]. In fact, the P127T change and the T118V/A128V double mutation in D/*ayw3*, as well as the P127I/L residue in genotypes E and F, have been reported to be associated with reduced HBsAg reactivity [32,33]. Also, the more complex substitutions at positions 118, 127, and 128 in D/*ayw3* can explain the more pronounced sensitivity reduction compared to one substitution at position 127 with E/*ayw4* and F/*adw4*. Because the P127T substitution is common for D/*ayw3* and also occurs in genotype A subtype *adw3*, it may be that these subtypes can exhibit reduced antigenicity as well. In the case of E/*ayw4* and F/*adw4*, there is also the genotype specific T140S residue which has been postulated to cause conformational change of the ‘*a*’ determinant [19]. In fact, in one HBsAg assay there was a slightly reduced sensitivity (factor 2–3) with E/*ayw4* and F/*adw4* but not with D/*ayw3*. The other subtype specific variations, including the d/*y* and *r/w* alleles at position 122 and 160, and the subtype specific residues at positions T126I, N131T, N134T, F143Y, A159G, Y161F, V168A, and P178Q, appear not to be involved in reduced antigenicity in this study. The influence of genotype and subtype variability on the sensitivity of HBsAg assays in the literature is somewhat inconclusive. Some investigators report that HBV genotypes A-G can be recognized comparably by commercial HBsAg assays including genotype E [34–37]. In contrast, others found sensitivity differences between HBV genotypes: up to 10-fold differences in the sensitivity of three commercial assays [38]; lower binding of anti-HBs by a factor 2–3 to *ayw* and *adr* compared to the WHO reference *adw* [39]; one of 10 HBsAg kits failed to give positive results with genotype E at 0·2 IU/ml [35]; and reduced reactivity in monoclonal antibody binding studies for E/*ayw4* and D/*ayw3* [33]. The results of the 17 highly sensitive HBsAg assays in this study essentially confirm that HBsAg kits used in the EU and Japan detect HBV genotypes A-F with comparable sensitivity [34,35]. Moreover, the present study shows that a substantial number (20 test kits from around the world) had impaired sensitivity for genotypes D/*ayw3,* E/*ayw4* and F/*adw4*.

In addition to the genotypes/subtypes, there was sensitivity reduction by S gene mutants in the ICBS Clinical Panel. A total of 32 HBsAg assays showed significant sensitivity reduction with one to five HBsAg mutants. Three HBsAg mutants identified, i.e., M133L (sample #1125), T131I (sample #1010), and S143L (sample #1015), impacted the sensitivity of HBsAg assays in this study as found in previous studies [4,5,9,40]. The two other HBsAg mutations found, P105R (sample #1135) and Q101H (sample #1039), are outside the ‘*a*’ determinant and have not been reported thus far to affect the sensitivity of commercial HBsAg assays. Mutants S143L and Q101H could not be detected by some HBsAg test kits. In other HBsAg test kits, there was reduced sensitivity of different degrees (factor >2 to 100). Nevertheless, smaller sensitivity reductions in the twofold range yielded false negative results when close to the assays′ cutoff, i.e., at low HBsAg concentrations of 0·21–0·36 IU/ml in mutant samples #1125 (M133L), #1135 (P105R), and #1010 (T131I). This demonstrates, once again, the need for quantitative analysis of HBsAg sensitivity as well as the relevance of low detection thresholds.

It has been postulated that the main reason for the different recognition capabilities of HBsAg mutants is the assay format, i.e., utilization of monoclonal antibodies for capture and detection phase (mono/mono) compared to assays based on polyclonal antibody in the conjugate phase (mono/poly) [38]. Nevertheless, in this study, there were assays with comparable sensitivity for the HBV genotypes tested but with deficiencies in detection of HBsAg mutants, as well as assays with no sensitivity reduction for HBsAg mutants but vulnerable to HBV genotypes/subtypes. For instance, the Advia Centaur HBsAg and Ortho HBsAg assays which have mono/mono test design were weak in detecting mutant T143L but detected all HBV genotypes/subtypes comparably. On the other hand, the Immulite HBsAg assay had difficulties in detecting mutants M133L and P105R despite having a polyclonal detection phase [4]. Therefore, epitope recognition seems to be more significant for mutant detection than the assay format [9]. The worst scenario seems to be represented by those kits that were compromised in mutant and genotype/subtype detection indicating a mono/mono test design with deficiencies in detecting epitopes in loop1 and loop 2 of the ‘*a*’ determinant. Generally, HBsAg test kits that include multiple monoclonal antibodies in the capture phase together with a polyclonal conjugate phase seem to be the best choice to assure recognition of different HBsAg epitopes.

Specificity evaluated on a panel of 200 negative samples was at an acceptable level of ≥99·5% [8] for the majority of HBsAg test kits (*n* = 57) and revealed an effective balance between the assays sensitivity and specificity. Nevertheless, nine HBsAg assays showed slightly lower specificities in the range of 96·37–99·0%. Four assays were not supplied in sufficient quantity to enable specificity testing. It should be noted that the number of 200 negative samples might be too low for statistically firm conclusions. In order to prevent loss of blood donations due to poor assay specificity and to avoid serious operational difficulties because of high rates of initial reactive non-specificities, it is recommended to test for high assay specificity with a higher number of negative samples, e.g. >2000 or 5000 [8], from the target population.

In conclusion, ICBS has established well-characterized panels for assessing the performance of HBsAg assays including genotype/subtype dependent sensitivity. The evaluation of 70 HBsAg test kits in this study revealed significant variation in diagnostic efficacy. A relatively high number of assays, including all rapid tests, were of poor sensitivity rendering them unsuitable for HBsAg detection at low concentrations. Therefore, these assays cannot be recommended for use within a public health context, e.g., blood screening. Genetic variability in the S gene additionally impaired diagnostic efficacy. We hope therefore that the results of the study would draw attention to the variability of HBsAg test kits available on the market as well as encourage the blood transfusion services within the resource-challenged countries to, whenever possible, locally evaluate the assays to be introduced for blood screening for appropriate sensitivity and specificity or to seek for corresponding published information. In order to expand access to high quality of assays it is also advisable to use more than one routine batch for evaluation.

## Supporting information

**Table S1** ICBS HBsAg Clinical Panel

**Table S2** Analytical sensitivities of the 70 HBsAg test kits evaluated

**Table S3** Day delay in detection of HBsAg during seroconversion at various assays’ detection limits

**Table S4 Sensitivity of HBsAg assays in the ICBS HBsAg Clinical Panel and results for the samples which showed difficulties to be detected by some HBsAg test kits**
