# Supplementary material for: Performance evaluation of 70 hepatitis B virus (HBV) surface antigen (HBsAg) assays from around the world by a geographically diverse panel with an array of HBV genotypes and HBsAg subtypes
Source: Vox Sang. 2010 Apr;98(3p2):403–14. doi: 10.1111/j.1423-0410.2009.01272.x (PMC2860763; doi:10.1111/j.1423-0410.2009.01272.x)
Supplement: Supplementary file 1 [file vox0098-0403-SD1.pdf]

Additional Supporting Information may be found in the online version of this article. The supporting information is found in the following tables:

- **Table S1: ICBS HBsAg Clinical Panel**
- **Table S2: Analytical sensitivities of the 70 HBsAg test kits evaluated**
- **Table S3: Day delay in detection of HBsAg during seroconversion at various assays' detection limits**
- **Table S 4: Sensitivity of HBsAg assays in the ICBS HBsAg Clinical Panel and results for the samples which showed difficulties to be detected by some HBsAg test kits**

Table S1: ICBS HBsAg Clinical Panel

ICBS HBsAg sample #	Country of origin	HBV Genotype	HBsAg Subtype	ICBS HBsAg sample #	Country of origin	HBV Genotype	HBsAg Subtype
1001	Ivory Coast	E	*Ayw4*	1074	Brazil	A	*adw2*
1002	Ivory Coast	E	*Ayw4*	1075	Brazil	A	*adw2*
1003	Ivory Coast	E	*Ayw4*	1076	Brazil	A	*adw2*
1004	Ivory Coast	E	*Ayw4*	1077	Brazil	A	*adw2*
1005	Ivory Coast	E	*Ayw4*	1078	Brazil	A	*adw4*
1006	Egypt	D	*Adw2*	1079	Brazil	F	*adw4*
1007	Egypt	D	*adw2*	1080	Brazil	F	*adw4*
1008	Egypt	D	*ayw2*	1081	U.S.A.	A	*adw2*
1009	Tunisia	D	*ayw2*	1082	U.S.A.	C	*adr*
1010	Tunisia	D	*ayw2*	1083	U.S.A.	A	*adw2*
1011	Tunisia	D	*ayw2*	1084	U.S.A.	C	*adr*
1012	Tunisia	A	*adw2*	1085	U.S.A.	D	*ayw2*
1013	Tunisia	D	*ayw2*	1086	U.S.A.	B	*adw2*
1014	Tunisia	D	*ayw2*	1087	U.S.A.	A	*adw2*
1015	Tunisia	D	*ayw2*	1088	U.S.A.	A	*adw2*
1016	Tunisia	A	*adw2*	1089	Vietnam	B	*ayw1*
1017	South Africa	D	*adw2*	1090	Vietnam	C	*adr*
1018	South Africa	A	*ayw2*	1091	Vietnam	B	*ayw1*
1019	Jordan	D	*ayw2*	1092	Vietnam	B	*ayw1*
1020	Jordan	D	*ayw2*	1093	Vietnam	B	*ayw1*
1021	Jordan	A	*adw2*	1094	Vietnam	C	*adr*
1022	Jordan	D	*ayw2*	1095	Vietnam	B	*ayw1*
1023	Jordan	D	*ayw2*	1096	Vietnam	B	*adw2*
1024	Jordan	D	*ayw2*	1097	Vietnam	B	*ayw1*
1025	Jordan	D	*ayw2*	1098	Vietnam	B	*adw2*
1026	Jordan	D	*ayw2*	1099	Vietnam	C	*adr*
1027	Jordan	A	*adw2*	1100	Vietnam	B	*ayw1*

1028	Jordan	D	*ayw4*	1101	Vietnam	B	*ayw1*
1029	Ivory Coast	E	*ayw4*	1102	Vietnam	B	*ayw1*
1030	Ivory Coast	E	*ayw4*	1103	Vietnam	B	*Ayw1*
1031	Ivory Coast	E	*ayw4*	1104	Vietnam	C	*Adr*
1032	Ivory Coast	E	*ayw4*	1105	Vietnam	B	*Ayw1*
1033	Ivory Coast	E	*ayw4*	1106	Vietnam	B	*Ayw1*
1034	Ivory Coast	E	*ayw4*	1107	Vietnam	B	*Ayw1*
1035	Ivory Coast	E	*ayw4*	1108	Vietnam	B	*Ayw1*
1036	Ivory Coast	A	*ayw1*	1109	Vietnam	B	*Ayw1*
1037	Ivory Coast	A	*ayw1*	1110	Vietnam	B	*Ayw1*
1038	Ivory Coast	E	*ayw4*	1111	U.S.A.	B	*Adw2*
1039	Ivory Coast	E	*ayw4*	1112	U.S.A.	D	*Ayw2*
1040	Ivory Coast	A	*ayw1*	1113	U.S.A.	F	*Adw4*
1041	Ivory Coast	A	*ayw1*	1114	U.S.A.	B	*Adw2*
1042	Ivory Coast	E	*ayw4*	1115	U.S.A.	B	*Adw2*
1043	Ivory Coast	E	*ayw4*	1116	U.S.A.	C	*Adr*
1044	Ivory Coast	E	*ayw4*	1117	U.S.A.	A	*Adw2*
1045	Ivory Coast	E	*ayw4*	1118	U.S.A.	A	*Adw2*
1046	Ivory Coast	E	*ayw4*	1119	U.S.A.	D	*Ayw3*
1047	Ivory Coast	E	*ayw4*	1120	U.S.A.	B	*Adw2*
1048	Ivory Coast	E	*ayw4*	1121	U.S.A.	B	*Adw2*
1049	Ivory Coast	E	*ayw4*	1122	U.S.A.	C	*Adr*
1050	Ivory Coast	E	*ayw4*	1123	U.S.A.	A	*Adw2*
1051	Ivory Coast	A	*ayw1*	1124	U.S.A.	D	*Ayw3*
1052	Ivory Coast	E	*ayw4*	1125	U.S.A.	B	*Adw2*
1053	Ivory Coast	E	*ayw4*	1126	U.S.A.	C	*Adr*
1054	Ivory Coast	E	*ayw4*	1127	U.S.A.	D	*Ayw3*
1055	Ivory Coast	E	*ayw4*	1128	U.S.A.	A	*Ayw1*
1056	Ivory Coast	E	*ayw4*	1129	U.S.A.	B	*Ayw1*
1057	Ivory Coast	E	*ayw4*	1130	U.S.A.	E	*Ayw4*
1058	Brazil	C	*adw2*	1131	U.S.A.	C	*Adr*
1059	Brazil	A	*adw2*	1132	U.S.A.	B	*Adw2*

1060	Brazil	D	*ayw2*	1133	U.S.A.	B	*Adw2*
1061	Brazil	A	*adw2*	1134	U.S.A.	A	*Adw2*
1062	Brazil	A	*adw2*	1135	U.S.A.	B	*Adw2*
1063	Brazil	D	*ayw3*	1136	Brazil	D	*Ayw2*
1064	Brazil	A	*adw2*	1137	Brazil	A	*Adw2*
1065	Brazil	F	*adw4*	1138	Brazil	A	*Adw2*
1066	Brazil	D	*ayw2*	1139	Brazil	A	*Adw2*
1067	Brazil	A	*adw2*	1140	Brazil	F	*Adw4*
1068	Brazil	D	*ayw2*	1141	Brazil	F	*Adw4*
1069	Brazil	D	*ayw2*	1142	Brazil	F	*Adw4*
1070	Brazil	F	*adw4*	1143	Brazil	D	*Ayw2*
1071	Brazil	F	*adw4*	1144	Brazil	F	*Adw4*
1072	Brazil	F	*adw4*	1145	Brazil	F	*Adw4*
1073	Brazil	B	*adw2*	1146	Brazil	F	*Adw4*

Table S2: Analytical sensitivities of the 70 HBsAg test kits evaluated

HBsAg test kit [1] and manufacturers	Catalogue No. [2]	Test format	PEI HBsAg *ad* Stand-ard	ICBS HBsAg Quantitative Panel (sample #, genotype/subtype, country of origin) #220 A/*adw2* Jordan	#546 B/*ayw1* Vietnam	#570 B/*adw2* Vietnam	#516 C/*adr* Vietnam	#93 D/*ayw2* Tunisia	#318 D/*ayw3* Brazil	#246 E/*ayw4* Ivory Coast	#713 F/*adw4* Brazil Manaus
			Detection limits (IU/mL [3])								
Prism HBsAg; Abbott GmbH & Co, KG	3A47-48	ChLIA	0.021	0.017	0.013	0.013	0.014	0.013	0.016	0.013	0.020
Enzygnost HBsAg 5.0 [4] Siemens Healthcare Diagnostics Products GmbH [5]	OQPW	EIA	0.021	0.016	0.045	0.036	0.044	0.016	0.041	0.033	0.041
Advia Centaur HBsAg Siemens Healthcare Diagnostics Inc. [6]	03393362	ChLIA	0.023	0.031	0.032	0.024	0.034	0.034	0.040	0.016	0.028
Elecsys HBsAg II Roche Diagnostics GmbH	04687787	ECLIA	0.023	0.058	0.046	0.046	0.041	0.034	0.049	0.029	0.051
Murex HBsAg Version 3 Abbott Murex Biotech, Ltd.	GE34	EIA	0.025	0.033	0.032	0.037	0.031	0.029	0.035	0.022	0.052
DS-IFA-HBsAg [4] RPC Diagnostic Systems	B-1152	EIA	0.035	0.113	0.079	0.080	0.108	0.067	0.076	0.036	0.110
Architect HBsAg Abbott GmbH & Co, KG	6C36	CLMIA	0.038	0.031	0.031	0.031	0.031	0.025	0.044	0.026	0.031
Ortho Antibody to HBsAg ELISA TS 3 Ortho-Clinical-Diagnostics, Inc.	931802	EIA	0.040	0.104	0.180	0.143	0.094	0.064	0.094	0.063	0.100
AxSYM HBsAg V2 Abbott GmbH & Co, KG	7A40-22	MEIA	0.042	0.065	0.033	0.029	0.033	0.037	0.072	0.052	0.041
Hepascan HBsAg Immunoenzyme TS BioService Borovsk	0798	EIA	0.042	0.065	0.050	0.057	0.062	0.056	0.058	0.027	0.044
EIAgen HBsAg Kit Adaltis Italia S.p.A.	071000E	EIA	0.043	0.041	0.039	0.029	0.060	0.036	0.031	0.018	0.053
ETI-MAK 4 HBsAg EIA Diasorin S.p.A.	CE0459	EIA	0.049	0.034	0.037	0.057	0.034	0.034	0.030	0.021	0.047
Monolisa HBsAg Ultra Bio-Rad Laboratories, Inc.	72348	EIA	0.051	0.057	0.048	0.048	0.055	0.033	0.036	0.017	0.059
Vectohep B - HBs-antigen Vector Best Novosibirsk	D-0555	EIA	0.057	0.083	0.046	0.045	0.064	0.040	0.045	0.017	0.077
Surase B-96 GBI General Biologicals Corp.	4SGE3	EIA	0.072	0.125	0.114	0.133	0.207	0.138	0.193	0,207	0.174

Cobas Core HBsAg II EIA [7)]<br>Roche Diagnostics GmbH	2075305 190	EIA	0.082	0.063	0.068	0.067	0.076	0.076	0.072	0.033	0.064
Hepanostika HBsAg Uni-Form II Microelisa system;<br>BioMérieux	280251, 280252	EIA	0.088	0.075	0.061	0.056	0.082	0.054	0.076	0.032	0.105
HBsAg ELISA Kit<br>Shenzhen Huakang Co., Ltd.	2101-01296	EIA	0.093	0.104	0.211	0.281	0.169	0.169	**4.9**	**1.0**	**0.86**
Immulite 2000 Hepatitis B Surface Ag<br>Siemens Healthcare Diagnostics Inc. [8)]	LKHB	LEIA	0.105	0.072	0.048	0.058	0.087	0.054	0.054	0.024	0.070
bioelisa HBsAg colour<br>biokit S.A.	3000-1130	EIA	0.119	0.102	0.098	0.108	0.159	0.095	0.158	0.103	0.093
HBs-antigen-DS<br>Medical-Biological-Union, Novosibirsk	D-063 (R)	EIA	0.119	0.169	0.133	0.147	0.141	0.125	0.146	0.100	0.180
Diagnostic Kit for Hepatitis B Surface Antigen<br>(ELISA) Beijing BGI-GBI Biotech, Ltd.	S20023027	EIA	0.129	0.133	0.125	0.129	0.370	0.122	**6.5**	**1.3**	**0.93**
Diagnostic Kit for Hepatitis B Surface Antigen<br>(ELISA) Shanghai Rongsheng Biotech, Ltd.	S10950045	EIA	0.130	0.241	0.445	0.408	0.292	0.401	**5.0**	**1.7**	**1.2**
Advanced HBsAg ELISA Test Kit<br>InTec Products, Inc.	S10910148	EIA	0.138	0.096	0.133	0.142	0.131	0.142	**2.3**	**0.49**	**0.54**
Akvapast Elisa HBsAg<br>Akvapast, St. Petersburg	No	EIA	0.139	0.212	0.153	0.130	0.103	0.160	0.074	0.118	0.094
Eliscan HBsAg (Microwell ELISA)<br>Ranbaxy Laboratories Ltd.	CD-11-32-18	EIA	0.149	0.191	0.188	0.137	0.202	0.217	**0.428**	0.230	0.101
HBsAg One Step<br>MBS SRL Medical Biological Services	1019	EIA	0.154	0.193	0.116	0.250	0.163	0.274	0.283	**0.57**	**0.40**
Hepatitis B Virus Surface Antigen (HBsAg) ELISA;<br>Beijing Wantai Biological Pharmacy Enterprise Co.,<br>Ltd.	WB-2296	EIA	0.166	0.228	0.308	0.360	0.314	0.367	**7.0**	**3.3**	**6.0**
Rapid HBsAg ELISA Kit<br>Sino American Biotechnology Co., Ltd.	DE02102	EIA	0.179	0.360	0.412	0.530	0.270	0.435	**9.5**	**4.0**	**3.9**
Equi-HBsAg<br>Equipar Diagnostici SRL	1910BG	EIA	0.184	0.305	0.197	0.164	0.230	0.178	0.160	0.054	0.121
EasiLisa HBsAg<br>Nubenco Diagnostics	A-EIA-116	EIA	0.189	0.313	0.503	0.589	0.441	0.614	**6.8**	**3.5**	**2.5**
Diagnostic Kit for Hepatitis B Surface Antigen<br>(ELISA); Shanghai Feilong Medical Diagnostic<br>Articles Co., Ltd.	S10980066	EIA	0.218	0.215	0.309	0.290	0.226	0.196	0.263	0.143	0.238
Kehua HBsAg<br>Shanghai Kehua Bioengineering Co., Ltd.	KH-T-01	EIA	0.227	0.311	0.506	0.533	0.271	0.478	0.200	0.309	0.291
Livzon HBsAg ELISA Kit<br>Livzon Group Reagent Factory	S10910154	EIA	0.267	0.350	0.562	0.569	0.882	0.688	**8.5**	**5.3**	**6.3**
Umelisa HBsAg Plus	D0007-10	EIA	0.269	0.250	0.359	0.541	0.317	0.575	**5.2**	**1.6**	**0.51**

Suma Centro de ImmunoEnsayo											
Diagnostic Kit for Hepatitis B Surface Antigen (ELISA); Zhengzhou Lifeclone Co., Ltd	S20013011	EIA	0.286	0.284	0.488	0.555	0.418	0.428	**4.1**	**1.2**	**0.84**
IFA-HBsAg Mikrogen, FGUP NPO, Moskva	002207/01-2003	EIA	0.298	0.376	0.335	0.402	0.282	0.433	0.218	0.256	0.458
IFA-HBsAg-antigen Pasteur Institute, St. Petersburg	No	EIA	0.350	0.351	0.270	0.257	0.258	0.212	0.254	0.281	0.304
Diagnostic Kit for HBsAg (ELISA) Autobio Zhengzhou Co., Ltd.	E0315-2	EIA	0.385	0.346	0.600	0.625	0.495	0.401	**4.2**	**1.0**	**0.95**
HBsAg ELISA Zhongshan Bio-Tech Co., Ltd	S10910123	EIA	0.474	0.598	0.961	0.970	0.848	0.568	**2.7**	**2.6**	**2.0**
Diagnostic Kit for Hepatitis B Surface Antigen (ELISA) Henan Lily Bioengineering Co., Ltd.	S20013011	EIA	0.590	0.727	1.326	1.348	1.083	1.124	**8.6**	**2.7**	**1.5**
Diagnostic Kit for HBsAg (ELISA) Beijing GWK Medical Biotechnology Co., Ltd.	No	EIA	0.638	0.753	1.359	1.493	1.067	1.274	**9.8**	**4.2**	**3.6**
BGH HBsAg ELISA Kit BGH Biochemical Co., Ltd.	S19980088	EIA	0.853	0.653	3.728	3.738	3.717	3.754	**13.0**	**12.0**	**12.0**
Diagnostic Kit for Hepatitis B Surface Antigen (ELISA) Beijing Four Rings Co., Ltd.	400038	EIA	1.0	1.9	4.3	4.6	2.9	4.4	**10.7**	**7.2**	**10.0**
HBsAg EIA VedaLab	1011	EIA	1.2	0.69	1.1	1.7	2.0	2.3	**5.1**	**5.4**	**3.2**
LG HBsAg ELISA LG Life Sciences, Ltd.	LGL-HBD 100-EN1-0310	EIA	1.4	1.2	0.97	1.24	0.45	0.64	0.85	1.24	0.48
Radiopreparat ELISA-HBsAg Radiopreparat Enterprise	No	EIA	2.1	4.4	3.1	2.4	5.9	2.8	2.2	3.4	3.1
SD BioLine HBsAg (One Step HBsAg Test) Standard Diagnostics, Inc.	01FK10	Rapid	1.7	1.5	1.5	1.5	1.5	1.5	1.5	1.5	1.5
One Step HBsAg Cassette Test Cypress Diagnostics	142-310	Rapid	1.7	4	4	4	4	4	4	4	4
HBsAg Dipstick One Step HBsAg Test World of Health Biotech Company	No	Rapid	1.7	4	4	4	4	4	**>4**	**>4**	**>4**
Determine HBsAg Inverness Medical Japan Co., Ltd..	7D25-13	Rapid	2.3	4	1	4	4	4	4	4	4
Quick Chaser HBsAg Mizuho Medy Co., Ltd.	61040	Rapid	2.3	4	4	4	4	4	4	4	4
Acon HBsAg One Step Diagnostic Test Strip Acon Laboratories, Inc.	IHBsg-301	Rapid	2.3	4	>4	>4	4	>4	>4	>4	>4
Erba LISA Hepatitis B ERBA Diagnostics Mannheim GmbH	HEPB	EIA	2.7	2.7	3.1	2.8	2.9	1.6	2.3	1.1	2.3
Diagnostic Kit for Hepatitis B Surface Antigen (ELISA) Shanghai Hua Tai Biotech, Ltd.	S10940048	EIA	3.4	3.9	7.9	7.9	8.4	8.4	8.0	8.1	8.0

Microscreen HBsAg ELISA Test Kit<br>Span Diagnostics, Ltd.	25967A	EIA	3.9	2.4	2.7	2.0	3.7	1.3	2.2	0.81	2.1
Hepalisa (HBsAg)<br>J. Mitra & Co., Ltd.	IRO20096	EIA	4.0	7.7	8.0	8.0	8.0	4.1	8.0	5.7	7.2
One Step HBsAg Dipstick Test<br>Newmarket Laboratories, Ltd. [9)]	No	Rapid	>2.3	>4	4	>4	4	>4	>4	>4	>4
Assure HBsAg Rapid Test<br>MP Biomedicals, Ltd.	43471-020	Rapid	>2.3	>4	>4	>4	>4	>4	>4	>4	>4
One Step Bioline Hepatitis B Surface Antigen Test<br>Strip; Pacific Biotech Co., Ltd.	No	Rapid	>2.3	>4	>4	>4	>4	>4	>4	>4	>4
Hepacard One Step Rapid Visual Test<br>J. Mitra & Co., Ltd.	HB010100	Rapid	>2.3	>4	>4	>4	>4	>4	>4	>4	>4
HepaScan HBsAg Cassette Type / Strip Type<br>YD Diagnostics	IM702-50S,<br>IM702-100	Rapid	>2.3	>4	>4	>4	>4	>4	>4	>4	>4
HBsAg (WB)<br>David & Tom Biotechnology Co., Ltd.	No	Rapid	>2.3	>4	>4	>4	>4	>4	>4	>4	>4
Hep-Check-1-Strip (HBsAg)<br>VedaLab	2034	Rapid	>2.3	>4	>4	>4	>4	>4	>4	>4	>4
Hepatitis B Antigen (HBsAg) Cassette<br>Equipar Diagnostici SRL	30HBGDE-A5	Rapid	>2.3	>4	>4	>4	>4	>4	>4	>4	>4
i+LAB HBsAg Test<br>i+MED Laboratories Co., Ltd.	HB-13IL, FL0050	Rapid	>2.3	>4	>4	>4	>4	>4	>4	>4	>4
Quick HBsAg Test<br>Firmer Co., Ltd.	F3-C4	Rapid	>2.3	>4	>4	>4	>4	>4	>4	>4	>4
Hep-Check-1 (Mini Clip HBsAg)<br>VedaLab	2071	Rapid	>2.3	>4	>4	>4	>4	>4	>4	>4	>4
Hep-alert-B One step HBsAg Card Test<br>Ranbaxy Laboratories Ltd.	1638/97	Rapid	>2.3	>4	>4	>4	>4	>4	>4	>4	>4
HBsAg Line Test Device<br>Acon Biotech Co., Ltd.	CD-5-2362	Rapid	>2.3	>4	>4	>4	>4	>4	>4	>4	>4

Footnotes

Values indicating genotype dependent sensitivity reduction are in **bold.**

Chlia=Chemiluminescent immunoassay; CLMIA=Chemiluminescence magnetic microcorpuscle immunoassay; ECI=Enhanced chemiluminescence immunoassay; ECLIA=Electro chemiluminescence immunoassay; EIA=Enzyme immunoassay: heterogeneous methods (EIA, ELISA); LEIA=Luminescence enhanced enzyme immunoassays; MEIA=Microparticle enzyme immunoassay; Rapid=Rapid assay various methods.

[1] Test kits sorted according to their analytical sensitivity with the PEI HBsAg *ad* standard (IU/mL).

[2] Catalogue no. as far identifiable from the outer wrap of the kit box (No = catalogue no. not identifiable)

[3] Relative to the 2nd WHO International HBsAg standard (00/588).

[4] New version of the test available in the meantime with enhanced sensitivity

[5] Formerly Dade-Behring Marburg

[6] Formerly Bayer Health Care LLC

[7] Test kit no longer marketed

[8] Formerly DPC Bierman LA

[9] Now Lab21, Ltd.

Table S3: Day delay in detection of HBsAg during seroconversion at various assays' detection limits

HBV serocon version panel catalogue #	HBsAg assay detection limits (IU/mL [1]) 0.02 [2]	0.05	0.13	0.5	1.0	2.0	4.0
	Day delay in detection of HBsAg						
PHM902	0	6.1	12.4	21.4	26.0	30.6	35.2
PHM904	0	3.4	7.0	12.1	14.7	17.3	19.9
PHM911	0	4.4	9.0	15.4	18.7	22.1	25.4
PHM912	0	3.2	6.6	11.3	13.7	16.1	18.6
PHM919	0	3.4	7.0	12.1	14.7	17.3	19.9
PHM922	0	2.8	5.6	9.6	11.7	13.7	15.8
PHM926	0	3.8	7.8	13.5	16.4	19.4	22.3
PHM927	0	2.8	5.6	9.6	11.7	13.8	15.8
PHM928	0	2.1	4.3	7.4	9.0	10.6	12.2
PHM929	0	3.3	6.7	11.6	14.1	16.6	19.1
PHM930	0	2.4	4.8	8.4	10.2	12.0	13.8
PHM931	0	2.7	5.5	9.5	11.6	13.6	15.7
PHM932	0	4.4	9.0	15.5	18.8	22.1	25.5
PHM934	0	2.6	5.3	9.2	11.2	13.2	15.2
6271	0	2.2	4.4	7.5	9.1	10.7	12.3
6272	0	6.3	12.9	22.2	26.9	31.7	36.5
6273	0	2.1	4.4	7.5	9.1	10.7	12.3
6274	0	2.8	5.8	10.0	12.1	14.3	16.5
6275	0	4.3	8.8	15.2	18.4	21.7	25.0
11000	0	3.2	6.5	11.2	13.6	16.0	18.4
11001	0	3.4	6.8	11.7	14.3	16.8	19.3
11003	0	4.3	8.8	15.2	18.4	21.7	25.0
11005	0	7.2	14.6	25.1	30.6	36.0	41.4
11006	0	2.8	5.7	9.7	11.8	13.9	16.0
11007	0	6.5	13.2	22.7	27.6	32.5	37.3
11008	0	4.0	8.2	14.1	17.1	20.1	23.2
11009	0	1.6	3.4	5.9	7.1	8.4	9.7
11011	0	3.5	7.2	12.4	15.1	17.8	20.5
11012	0	3.3	6.8	11.7	14.2	16.8	19.3
11013	0	6.7	13.7	23.6	28.7	33.8	38.9
11016	0	2.7	5.5	9.4	11.4	13.5	15.5
11017	0	4.7	9.6	16.5	20.0	23.5	27.1
**Geomean [3]**	**0**	**3.5**	**7.1**	**12.2**	**14.8**	**17.5**	**20.1**
**Minimum [3]**	**0**	**1.6**	**3.4**	**5.9**	**7.1**	**8.4**	**9.7**
**Maximum [3]**	**0**	**7.2**	**14.6**	**25.1**	**30.6**	**36.0**	**41.4**

Footnotes

[1] IU/mL relative to 2nd WHO HBsAg international standard (00/588).

[2] Relative to the detection limit (IU/mL) of the most sensitive assays in Table S2.

[3] Geomean = geometric mean value; min/max = the smallest and largest number in the set of values.

Table S 4: **Sensitivity of HBsAg assays in the ICBS HBsAg Clinical Panel and results for the samples which showed difficulties to be detected by some HBsAg test kits**

	Clinical Sensitivity (n=146)	ICBS HBsAg Clinical Panel Samples (sample #, **genotype/*subtype***, HBsAg concentration in IU/mL)				
		#1125 **B/*adw2*** 0.21 IU/mL	#1135 **B/*adw2*** 0.22 IU/mL	#1010 **D/*ayw2*** 0.36 IU/mL	#1039 **E/*ayw4*** 72 IU/mL	#1015 **D/*ayw2*** >8000 IU/mL
**HBsAg test kit [1] and manufacturer**	**%**	**S/co [2], IU/mL [3] or reading [4]**				
Prism HBsAg<br>Abbott GmbH & Co, KG	100	7.81	11.50	77.05	685.71	485.48
Enzygnost HBsAg 5.0 [5]<br>Siemens Healthcare Diagnostics Products GmbH [6]	100	7.11	12.34	17.71	48.78	39.66
Advia Centaur HBsAg<br>Siemens Healthcare Diagnostics Inc. [7]	100	19.34	13.69	10.02	>1000.00	3.80
Elecsys HBsAg II<br>Roche Diagnostics GmbH	100	7.83	5.95	13.16	2553.00	2308.00
Murex HBsAg Version 3<br>Abbott Murex Biotech, Ltd.	100	1.81	2.49	9.11	>max	>max
DS-IFA-HBsAg [5]<br>RPC Diagnostic Systems	100	10.00	3.81	15.85	>max	57.52
Architect HBsAg<br>Abbott GmbH & Co, KG	100	0.21	0.22	0.36	71.99	>250
Ortho Antibody to HBsAg ELISA TS 3<br>Ortho-Clinical-Diagnostics, Inc.	100	1.34	3.60	9.03	>max	16.00
AxSYM HBsAg V2<br>Abbott GmbH & Co, KG	100	3.13	3.25	3.33	125.07	92.23
Hepascan HBsAg Immunoenzyme TS<br>BioService Borovsk	100	4.84	1.82	7.02	27.35	25.45
EIAgen HBsAg Kit<br>Adaltis Italia S.p.A.	100	1.04	1.32	5.25	65.64	68.14
ETI-MAK 4 HBsAg EIA<br>Diasorin S.p.A.	100	3.77	5.91	11.52	65.86	>max
Monolisa HBsAg Ultra<br>Bio-Rad Laboratories, Inc.	100	1.68	3.15	12.71	44.13	41.66

Vectohep B - HBs-antigen<br>Vector Best Novosibirsk	100	2.29	1.61	5.12	35.64	34.83
Surase B-96<br>GBI General Biologicals Corp.	98.63	**0.55**	**0.94**	2.94	102.78	102.78
Cobas Core HBsAg II EIA [8)]<br>Roche Diagnostics GmbH	100	1.76	76.08	22.33	76.08	75.71
Hepanostika HBsAg Uni-Form II Microelisa system<br>BioMérieux	100	1.83	1.62	3.49	48.27	50.16
HBsAg ELISA Kit<br>Shenzhen Huakang Co., Ltd.	99.32	**0.88**	1.08	1.73	22.96	67.50
Immulite 2000 Hepatitis B Surface Ag<br>Siemens Healthcare Diagnostics Inc. [9)]	98.63	**0.73**	**0.78**	5.80	1953.00	3808.00
bioelisa HBsAg colour<br>biokit S.A.	100	1.90	1.78	3.87	18.89	19.06
HBs-antigen-DS<br>Medical-Biological-Union, Novosibirsk	98.63	**0.75**	**0.59**	3.42	25.18	18.64
Diagnostic Kit for Hepatitis B Surface Antigen (ELISA)<br>Beijing BGI-GBI Biotech, Ltd.	100	1.22	1.48	7.00	19.18	59.12
Diagnostic Kit for Hepatitis B Surface Antigen (ELISA)<br>Shanghai Rongsheng Biotech, Ltd.	99.32	1.10	**0.66**	1.13	10.73	24.86
Advanced HBsAg ELISA Test Kit<br>InTec Products, Inc.	98.63	**0.52**	**0.52**	1.34	16.81	21.40
Akvapast Elisa HBsAg<br>Akvapast, St. Petersburg	99.32	4.50	**0.15**	1.02	10.38	11.29
Eliscan HBsAg (Microwell ELISA)<br>Ranbaxy Laboratories Ltd.	99.32	**0.90**	3.47	1.78	25.84	22.66
HBsAg One Step<br>MBS SRL Medical Biological Services	99.32	**0.92**	1.16	4.00	90.08	90.05
Hepatitis B Virus Surface Antigen (HBsAg) ELISA<br>Beijing Wantai Biological Pharmacy Enterprise Co., Ltd.	99.32	1.12	**0.94**	2.27	14.26	34.92
Rapid HBsAg ELISA Kit<br>Sino American Biotechnology Co., Ltd.	98.63	**0.39**	**0.54**	7.64	17.89	74.51
Equi-HBsAg<br>Equipar Diagnostici Societa a Responsabilita Limitata	99.32	1.30	**0.56**	3.23	52.23	47.53
EasiLisa HBsAg<br>Nubenco Diagnostics	98.63	**0.60**	**0.71**	1.02	8.49	11.64
Diagnostic Kit for Hepatitis B Surface Antigen (ELISA)<br>Shanghai Feilong Medical Diagnostic Articles Co., Ltd.	98.63	**0.51**	**0.46**	1.29	11.19	10.25
Kehua HBsAg<br>Shanghai Kehua Bioengineering Co., Ltd.	98.63	**0.48**	**0.90**	1.31	32.77	32.61

Livzon HBsAg ELISA Kit<br>Livzon Group Reagent Factory	98.63	**0.42**	**0.59**	6.91	4.13	27.98
Umelisa HBsAg Plus<br>Suma Centro de ImmunoEnsayo	99.32	5.14	**0.67**	2.10	38.90	38.90
Diagnostic Kit for Hepatitis B Surface Antigen (ELISA)<br>Zhengzhou Lifeclone Co., Ltd	97.95	**0.51**	**0.63**	**0.80**	18.15	25.14
IFA-HBsAg<br>Mikrogen, FGUP NPO, Moskva	98.63	**0.73**	**0.86**	1.15	11.56	>max
IFA-HBsAg-antigen<br>Pasteur Institute, St. Petersburg	99.32	8.32	**0.03**	1.54	23.61	25.64
Diagnostic Kit for HBsAg (ELISA)<br>Autobio Zhengzhou Co., Ltd.	98.63	**0.58**	**0.68**	1.12	19.47	29.42
HBsAg ELISA<br>Zhongshan Bio-Tech Co., Ltd	97.95	**0.30**	**0.38**	**0.82**	8.97	33.71
Diagnostic Kit for Hepatitis B Surface Antigen (ELISA)<br>Henan Lily Bioengineering Co., Ltd.	97.95	**0.57**	**0.36**	**0.88**	10.43	39.85
Diagnostic Kit for HBsAg (ELISA)<br>Beijing GWK Medical Biotechnology Co., Ltd.	97.95	**0.32**	**0.36**	**0.56**	4.48	18.73
BGH HBsAg ELISA Kit<br>BGH Biochemical Co., Ltd.	97.26	**0.13**	**0.11**	**0.55**	**0.95**	29.08
Diagnostic Kit for Hepatitis B Surface Antigen (ELISA)<br>Beijing Four Rings Co., Ltd.	97.95	**0.28**	**0.36**	**0.29**	2.77	25.89
HBsAg EIA<br>VedaLab	98.63	2.83	**0.47**	**0.58**	3.26	13.38
LG HBsAg ELISA<br>LG Life Sciences, Ltd.	98.63	**0.29**	**0.66**	1.03	43.57	38.25
Radiopreparat ELISA-HBsAg<br>Radiopreparat Enterprise	97.95	**0.73**	**0.59**	**0.65**	19.31	26.08
SD BioLine HBsAg (One Step HBsAg Test)<br>Standard Diagnostics, Inc.	97.95	**negative**	**negative**	**negative**	positive	positive
One Step HBsAg Cassette Test<br>Cypress Diagnostics	98.63	**negative**	**negative**	positive	positive	positive
HBsAg Dipstick One Step HBsAg Test<br>World of Health Biotech Company	97.26	**negative**	**negative**	**negative**	positive	**negative**
Determine HBsAg<br>Abbott Japan Co., Ltd	97.95	**negative**	**negative**	**negative**	positive	positive
Quick Chaser HBsAg<br>Mizuho Medy Co., Ltd.	97.95	**negative**	**negative**	**negative**	positive	positive
Acon HBsAg One Step Diagnostic Test Strip<br>Acon Laboratories, Inc.	97.95	**negative**	**negative**	**negative**	positive	positive

Erba LISA Hepatitis B<br>ERBA Diagnostics Mannheim GmbH	97.95	**0.0**	**0.0**	**0.19**	20.07	25.27
Diagnostic Kit for Hepatitis B Surface Antigen (ELISA)<br>Shanghai Hua Tai Biotech, Ltd.	97.26	**0.56**	**0.46**	**0.60**	**0.98**	22.52
Microscreen HBsAg ELISA Test Kit<br>Span Diagnostics, Ltd.	98.63	**0.70**	1.18	**0.62**	19.45	26.02
Hepalisa (HBsAg)<br>J. Mitra & Co., Ltd.	97.95	**0.18**	**0.65**	**0.13**	8.70	7.73
One Step HBsAg Dipstick Test<br>Newmarket Laboratories, Ltd. [10)]	97.95	**negative**	**negative**	**negative**	positive	positive
Assure HBsAg Rapid Test<br>MP Biomedicals, Ltd.	97.95	**negative**	**negative**	**negative**	**equivocal**	positive
One Step Bioline Hepatitis B Surface Antigen Test Strip<br>Pacific Biotech Co., Ltd.	97.95	**negative**	**negative**	**negative**	positive	positive
Hepacard One Step Rapid Visual Test<br>J. Mitra & Co., Ltd.	97.95	**negative**	**negative**	**negative**	positive	positive
HepaScan HBsAg Cassette Type / Strip Type<br>YD Diagnostics	97.95	**negative**	**negative**	**negative**	positive	positive
HBsAg (WB)<br>David & Tom Biotechnology Co., Ltd.	97.95	**negative**	**negative**	**negative**	positive	positive
Hep-Check-1-Strip (HBsAg)<br>VedaLab	97.26	**negative**	**negative**	**negative**	positive	**negative**
Hepatitis B Antigen (HBsAg) Cassette<br>Equipar Diagnostici Societa a Responsabilita Limitata	97.26	**negative**	**negative**	**negative**	positive	**negative**
i+LAB HBsAg Test<br>i+MED Laboratories Co., Ltd.	97.26 [†]	**negative**	**negative**	**negative**	positive	positive
Quick HBsAg Test<br>Firmer Co., Ltd.	97.26	**negative**	**negative**	**negative**	**negative**	positive
Hep-Check-1 (Mini Clip HBsAg)<br>VedaLab	97.26	**negative**	**negative**	**negative**	positive	**negative**
Hep-alert-B One step HBsAg Card Test<br>Ranbaxy Laboratories Ltd.	97.26 [†]	**negative**	**negative**	**negative**	positive	positive
HBsAg Line Test Device<br>Acon Biotech Co., Ltd.	94.52 [†]	**negative**	**negative**	**negative**	positive	positive

Footnotes

Negative and equivocal results are **bold**.

[1)] Test kits sorted according to their analytical sensitivity (see Table S2).

[2] S/co values ≥ 1 are positive, s/co values <1 are negative.

[3] IU/mL for the Architect HBsAg assay (Abbott), values ≥0.05 IU/mL are positive, values <0.05 IU/mL are negative.

[4] Rapid assay reading: positive, equivocal, negative.

† These rapid assays were negative in further samples not shown in this **Table** as explained in the results' section 3.3
